# A Cost-Benefit Analysis of Lipid Standardization in the United States

**Published:** 2011-10-15

**Authors:** Thomas J. Hoerger, John S. Wittenborn, Walter Young

**Affiliations:** RTI International; RTI International, Research Triangle Park, North Carolina; National Association of Chronic Disease Directors, Golden, Colorado

## Abstract

**Introduction:**

By improving lipid standardization, the Centers for Disease Control and Prevention's (CDC's) Lipid Standardization Program and Cholesterol Reference Method Laboratory Network have contributed to the marked reduction in heart disease deaths since 1980. The objective of this study was to estimate the benefits (ie, the value of reductions in heart disease deaths) and costs attributable to these lipid standardization programs.

**Methods:**

We developed a logic model that shows how the inputs and activities of the lipid standardization programs produce short- and medium-term outcomes that in turn lead to improvements in rates of cardiovascular disease and death. To calculate improvements in long-term outcomes, we applied previous estimates of the change in heart disease deaths between 1980 and 2000 that was attributable to statin treatment and to the reduction in total cholesterol during the period. Experts estimated the share of cholesterol reduction that could be attributed to lipid standardization. We applied alternative assumptions about the value of a life-year saved to estimate the value of life-years saved attributable to the programs.

**Results:**

Assuming that 5% of the cholesterol-related benefits were attributable to the programs and a $113,000 value per life-year, the annual benefit attributable to the programs was $7.6 billion. With more conservative assumptions (0.5% of cholesterol-related benefits attributable to the programs and a $50,000 value per life-year), the benefit attributable to the programs was $338 million. In 2007, the CDC lipid standardization programs cost $1.7 million.

**Conclusion:**

Our estimates suggest that the benefits of CDC's lipid standardization programs greatly exceed their costs.

## Introduction

Cholesterol awareness and control are important factors in reducing deaths from heart disease in the United States and are a key focus of health promotion and clinical practice (1). Age-adjusted death rates for heart disease have dropped in the United States from 1980 (412.1 per 100,000 population) to 2000 (257.6 per 100,000 population) and 2006 (200.2 per 100,000 population) (2,3). Using a model of the impact of various risk factors and treatments on heart disease deaths, Ford et al (4) and Capewell et al (5) attributed nearly one-third of the reduction in heart disease deaths between 1980 and 2000 to a reduction in the prevalence of high cholesterol and improved secondary prevention using statin drugs to control cholesterol in people with previous heart disease.

An important but sometimes overlooked contribution to improvements in cholesterol awareness and control has been provided by the Centers for Disease Control and Prevention's (CDC's) Lipid Standardization Program (LSP) and Cholesterol Reference Method Laboratory Network (CRMLN) ("lipid standardization programs" hereafter). The LSP is an accuracy-based program that defines benchmark reference methods and maintains stable pools of reference testing materials (6,7). Its standardization activities have supported epidemiologic studies that identified the role of cholesterol in heart disease and clinical research laboratories that conducted standardized clinical trials to test the effects of alternative treatments to reduce cholesterol. The CRMLN is a network of laboratories replicating the CDC reference methods to help manufacturers improve the accuracy of clinical testing methods (8). Because only a few manufacturers produce the diagnostic equipment and supplies used in cholesterol testing, facilitating accurate manufacturer calibration results in more accurate clinical testing nationwide (9).

As a cost-saving measure, in 2008 the National Heart, Lung, and Blood Institute (NHLBI) retracted its 50-year budgetary commitment to the LSP. Without the NHLBI funding, the LSP and CRMLN programs may not be able to continue in their present form. In response, the Cardiovascular Biomarker Standardization Steering Committee of the National Association of Chronic Disease Directors (NACDD) asked NACDD to conduct a cost-benefit study of the LSP. The objective of this study was to estimate the benefits and costs of the LSP and CRMLN. Results of the study may be used by policy makers to determine the value of lipid standardization.

## Methods

### Logic model development

**Figure 1.**
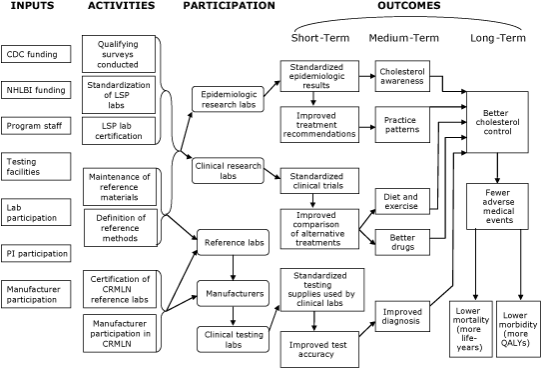
Lipid Standardization Program (LSP) and Cholesterol Reference Method Laboratory Network (CRMLN) logic model. Abbreviations: CDC, Centers for Disease Control and Prevention; NHLBI, National Heart, Lung, and Blood Institute; PI, principal investigator; QALY, quality-adjusted life-year.

We first developed a logic model for assessing the impact of the CDC lipid standardization programs (Figure 1). Logic models are a program evaluation tool used to graphically depict major elements and causality pathways of a program (10). The left side of the logic model lists the resources (inputs) necessary for program operation. The logic model then shows the major program activities; the participants involved with and affected by these activities; and the short-, medium-, and long-term outcomes of the programs.

The programs are currently supported by CDC and previously were jointly funded by NHLBI. We do not consider participation costs incurred by clinical laboratories, manufacturers, and research funding agents because participation in the programs is voluntary.

The fundamental activities of the programs are to define reference methods and maintain reference materials for total cholesterol, high-density lipoprotein cholesterol, low-density lipoprotein (LDL) cholesterol, and triglycerides. CDC uses a standardized testing protocol on well-characterized and uniform serum materials to eliminate potential reference bias and allow program participant results to be compared directly to the CDC reference results. The programs maintain a set of frozen serum pools that exhibit a wide range of lipid concentrations. Long-term maintenance of these pools is essential to ensure that reference values of these samples do not drift over time.

Using the reference measurement procedures and materials, the LSP conducts standardization of clinical laboratories involved in epidemiologic and clinical research. Establishing a long-term, accuracy-based reference allows results to be compared across different laboratories and over time, which is necessary when conducting multicenter clinical trials; comparing lipid measurement values to past values, such as the baseline period in clinical and epidemiologic research; or comparing lipid measurement values across clinical and epidemiologic research studies. Recommended accuracy goals for lipids and lipoprotein tests, which include both bias and imprecision, have been developed by the National Cholesterol Education Program (11-13). These accuracy goals are based on results obtained from the LSP.

The CRMLN uses the standardized reference methods and materials to certify a network of reference laboratories that seek to replicate the accuracy of the CDC laboratory. In turn, this network of laboratories certifies participating manufacturers and clinical testing laboratories. By calibrating manufacturers' equipment and testing methods against the CDC reference standards, the CRMLN increases the accuracy of all tests conducted using these supplies, even for clinical laboratories that do not participate in the program. This system allows for accurate comparison of test results to the clinical practice guidelines established by the Adult Treatment Panel III (14).

The programs' inputs, activities, and participants are intended to produce the following short-term outcomes:

- Standardized epidemiologic results, which lead to improved treatment recommendations.
- Standardized clinical trials, which lead to improved comparisons of alternative treatments.
- Standardized routine testing methods, which lead to the improved cholesterol test accuracy necessary for improved diagnosis.

The short-term outcomes — in combination with nonprogram factors — lead to the following medium-term outcomes for patients and health care providers: increased cholesterol awareness, better practice patterns, improved diet and exercise, better drugs, and improved diagnosis of patients with high cholesterol.

The medium-term outcomes combine to produce better cholesterol control, which improves patient health outcomes in the long term by reducing medical events and lowering rates of cardiovascular disease and death. The true benefits of the lipid standardization programs arise from improvements in these long-term patient outcomes.

### Quantifying outcomes

It is difficult to quantify precisely the effect of the lipid standardization programs on these short-, medium-, and long-term outcomes. For most of the outcomes, standardization of lipid measures is a prerequisite that can support and promote — but does not by itself guarantee — improved public health outcomes. For example, standardization supports clinical trials of new cholesterol-lowering therapies, but development of new therapies also depends on research and development efforts, technologic breakthroughs, and careful clinical testing.

Because we cannot directly attribute outcome changes to program operations, our approach is to investigate improvements in each short- and medium-term outcome during recent years. We discuss qualitatively how the standardization efforts of CDC programs may have facilitated the improvement, but we do not attempt to estimate precisely the share of each improvement that is attributable to the programs. We also do not place a dollar value on the benefits of improvements in short- and medium-term outcomes because the true benefits to patients are associated with improvements in long-term outcomes.

To determine the improvements in long-term outcomes, we applied previous estimates of the change in heart disease deaths between 1980 and 2000 that was attributable to treatment with statins and the overall reduction in total cholesterol during the period. Ford et al (4) and Capewell et al (5) estimated that these cholesterol-related changes prevented or postponed more than 111,000 deaths and saved 1.35 million life-years in 2000 (Table 1).

We considered alternative assumptions about the share of deaths prevented or postponed and life-years saved that were attributable to the lipid standardization programs. We asked experts to estimate the percentage of lipid reduction during the period that was attributable to the lipid standardization programs. The 4 experts work on cardiovascular disease in various settings (1 in a university hospital, 2 in private clinical laboratories, and 1 in the National Institutes of Health). The experts were asked to base their estimates on information on the reduction in CHD deaths between 1980 and 2000 and the estimate (4) that 24% of the reduction was due to lower cholesterol levels. They were told that lipid standardization was potentially one of many factors contributing to lower lipid levels. The experts' median estimate of the share of lipid reduction attributable to lipid standardization was 5%, but the estimates ranged widely (from 2%-3% to >50%). We also considered more conservative estimates of 0.5% and 1%.

To estimate the dollar value of improvements in life expectancy, we applied alternative estimates of the value of a life-year gained. Setting a dollar value on life-years gained is controversial. In the health economics literature, a value of $50,000 per quality-adjusted life-year (QALY) is sometimes used to assess the cost-effectiveness of interventions, but the conceptual basis for this benchmark is debatable (15). A recent study suggests that society's willingness to pay for health improvements is at least $113,000 per QALY in the United States (16). For regulatory purposes in cost-benefit analyses of environmental issues, the Environmental Protection Agency (EPA) sets the value of a statistical life-year at $300,000. The EPA estimate is based on an estimate of $4.8 million in 1990 dollars (17) for 1 statistical life that was calculated using wage differentials for risky jobs in the labor market. To convert this value into a value for a statistical life-year, we multiplied $4.8 million by 1.49, the gross domestic product inflator for 2008 relative to 1990. We assumed that the resulting value of a statistical life-year in 2008 represented the net present value of a stream of constant statistical life-year payments received over 40 years (the approximate remaining life expectancy of a 45-year-old adult) with a real discount rate of 3%. Solving for the value of a statistical life-year yields a value of $300,000 per life-year.

We calculated the dollar benefits of the life-years gained attributable to the lipid standardization programs under alternative assumptions about the life-years attributable to the LSP and CRMLN and the value of a life-year gained. We then compared the dollar benefits of the programs to the cost of the LSP and CRMLN in fiscal year 2008, $1.7 million.

## Results

As detailed in the Appendix, we found suggestive evidence linking the LSP and CRMLN to improved short- and medium-term outcomes. Program data indicate that laboratories participating in the programs achieve high levels of accuracy. This accuracy supports standardized epidemiologic results, standardized clinical trials, and standardized testing methods used by clinical laboratories, which can lead to improved test accuracy.

Assuming the median expert panel estimate of 5% of the cholesterol-related benefits attributable to the programs and a value per life-year of $113,000, the benefits attributable to the programs are estimated to be $7.6 billion (Table 2). Assuming the most conservative estimates (0.5% of the cholesterol-related benefits attributable to the programs and a $50,000 value per life-year), the benefits attributable to the programs are estimated to be $338 million annually.

**Figure 2.**
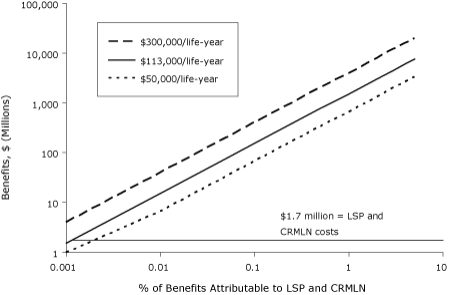
Benefits of life-years gained from the Lipid Standardization Program (LSP) and Cholesterol Reference Method Laboratory Network (CRMLN). Cost of the programs was $1.7 million per year.

We conducted a sensitivity analysis to examine how the estimated benefits change under alternative assumptions about the percentage attributable to the LSP and CRMLN (Figure 2) in comparison to this cost. Both the percentage attributable and the benefits and costs are presented using logarithmic scales to preserve the true linear relationship between the benefit and the percentage attributable and to allow the benefits and costs to be shown in the same figure (otherwise, the benefit at 5% attributable would completely dwarf the cost of the programs). Even assuming that the LSP and CRMLN are responsible for only 0.01% of the improvement in life-years attributable to cholesterol-related factors, the benefits of the programs substantially exceed their costs.

**Table:** 

**% of Benefits Attributable to LSP and CRMLN**	**Value per Life-Year, $**		
	**50,000**	**113,000**	**300,000**
0.001	1,000,000	1,526,856	4,053,600
0.010	6,756,000	15,268,560	40,536,000
0.050	33,780,000	76,342,800	202,680,000
0.100	67,560,000	152,685,600	405,360,000
0.500	337,800,000	763,428,000	2,026,800,000
1.000	675,600,000	1,526,856,000	4,053,600,000
2.000	1,351,200,000	3,053,712,000	8,107,200,000
3.000	2,026,800,000	4,580,568,000	12,160,800,000
4.000	2,702,400,000	6,107,424,000	16,214,400,000
5.000	3,378,000,000	7,634,280,000	20,268,000,000

## Discussion

Our estimates suggest that the benefits of CDC's lipid standardization programs greatly exceed their costs. Deaths from heart disease fell dramatically between 1980 and 2000 (2,3), and this reduction has driven improvements in overall life expectancy. The improvement in life-years has high dollar value. A significant share of the improvement in heart disease deaths is due to cholesterol-related factors, including the overall reduction in total and LDL cholesterol and the use of statin drugs for secondary and primary prevention (4,5). To the extent that some of the improvement in these cholesterol-related factors is due to CDC's lipid standardization programs, the programs have large dollar benefits because the overall dollar value of the increase in life-years from heart disease — and the share of the increase that is attributable to cholesterol-related factors — is so large. Put another way, the overall benefits of cholesterol reduction are so large that there is plenty of credit to go around to lipid standardization programs and other factors (eg, research and development efforts, technological breakthroughs, careful clinical testing) affecting cholesterol. Even if the LSP and CRMLN receive only a small share of the credit, the programs' benefits exceed their costs.

Our analysis has several potential limitations. First, our estimates could implicitly overestimate the value of the improvements in life expectancy stemming from reductions in heart disease deaths; however, this does not appear to be the case. Improvements in heart disease deaths are clearly documented in national life expectancy and cause-of-death data. Less evidence exists on the value of this improvement, but a study by Murphy and Topel (18) provides context for our estimates. They estimated that the reduction in deaths from heart disease has increased the value of life by about $1.5 trillion per year since 1970. The Capewell et al study (5) — which is the basis for our estimates — found that 3.15 million life-years were gained in 2000 from all reductions in heart disease deaths between 1980 and 2000. When this gain is valued at $50,000, $113,000, and $300,000 per life-year, the total benefits in 2000 would be $155 billion, $350 billion, and $930 billion, respectively. Thus, the overall benefit from reduction in heart disease deaths given by Murphy and Topel is even greater than the underlying heart disease benefit in our analysis.

Second, our analysis could attribute too much of the gain in life-years resulting from reductions in heart disease deaths to cholesterol-related factors. We relied on studies by Ford et al (4) and Capewell et al (5), which appear to provide the most comprehensive decomposition of the gain in life-years that are attributable to specific factors.

Third, our estimate depends on the share of lipid reduction that is attributable to the lipid standardization programs, and this parameter was not precisely measured. Although the parameter was based on the opinion of experts familiar with the programs, these experts noted that the parameter was difficult to estimate. Even when we included more conservative parameters, the benefits attributable to the programs were still sizeable. Because this is probably the most important potential limitation of our study, it is worth considering additional alternative estimates. The sensitivity analysis shows how the benefits change with the percentage attributable to the LSP and CRMLN for values ranging from 0.001% to 5%. Even when the LSP and CRMLN are responsible for a smaller share of the reduction in life-years attributable to cholesterol-related factors than our expert panel estimated, the benefits of the programs substantially exceed their costs.

Fourth, our estimate of the benefits attributable to the programs is based on the estimated effects of the programs on cholesterol between 1980 and 2000. It is not clear whether standardization would suffer today and in the future if the LSP and CRMLN ceased to exist. One might expect that test values would drift away from true values over time, but it seems unlikely that bias levels would return to their 1980 levels. This limitation can be assessed using Figure 2. Suppose that 1% of the improvement in cholesterol-related factors is due to LSP and CRMLN effects between 1980 and 2000. If 10% of this improvement would be lost if the programs ceased to exist, then the lost benefit would equal 0.1% of the overall cholesterol-related effects. The programs' benefits would still substantially exceed their costs.

Fifth, our estimates of the improvements in heart disease deaths that are attributable to cholesterol-related factors are based on studies for the period between 1980 and 2000. If more recent data were available, the number of life-years saved by cholesterol-related factors would likely increase because heart disease death rates have continued to fall since 2000.

Sixth, assigning a dollar value to life-years saved is controversial, and when a value is assigned, debate remains about the actual value to set. Nevertheless, the estimated benefit from the lipid standardization programs remains high even if life-years are valued at $50,000 per year.

Finally, our analysis does not include health care cost offsets or increases associated with the reduction in heart disease deaths. In principle, it might be possible to model spending on heart disease for individual patients; however, the level of modeling necessary is beyond the scope of this study.

This is the first study attempting to quantify the benefits of CDC's lipid standardization programs. At least 1 study has assessed the benefits of improving the accuracy of other clinical tests. Gallaher et al (19) estimated that systematic errors on calcium tests could increase the costs of follow-up testing and procedures by $60 million to $199 million per year. Although that study applied different methods and examined a different test than our study, it provides support for our general finding that improving test accuracy may have substantial financial benefits.

As noted in the introduction, the NHLBI discontinued its funding of the LSP in 2008, raising questions about the program's future. Our estimates provide evidence of the benefits and costs of the CDC lipid standardization programs that may help policy makers decide whether to continue funding the programs.

## Figures and Tables

**Table 1:** Deaths Prevented or Postponed and Life-Years Gained Attributable to Cholesterol-Related Factors, 2000^a^

**Factor**	Deaths Prevented or Postponed	Life-Years Gained
Statin treatment	28,785	249,125
Reduction in the prevalence of high cholesterol	82,800	1,102,100
Total	111,585	1,351,225

a Source: Capewell et al (5). Data from Ford et al (4) were used to calculate the deaths prevented or postponed and life-years gained that were attributable to treatment with statins.

**Table 2:** Benefits of Life-Years Gained From the Lipid Standardization Program (LSP) and Cholesterol Reference Method Laboratory Network (CRMLN)^a^

**Estimates**		Benefits, $ (Millions)		
**% Attributable to the LSP and CRMLN**	Life-Years Gained	$50,000 per Life-Year	$113,000 per Life-Year	$300,000 per Life-Year
0.5	6,756	338	763	2,027
1	13,512	676	1,527	4,054
5	67,561	3,378	7,634	20,268

a Benefits calculated as the share of cholesterol-related benefits attributable to the programs multiplied by the share of life-years gained that is attributable to cholesterol-related factors multiplied by the value of a life-year.

## Appendix

The following is an excerpt from: Hoerger TJ, Wittenborn JS, Couper S. Lipid standardization program: cost-benefit analysis: final report. Research Triangle Park (NC): RTI International; 2010.

### 3.1 Short-Term Outcomes: Improvements in Laboratory Standardization for Cholesterol Testing

The immediate outcome of improved lab standardization and manufacturer certification is an increase in the accuracy of cholesterol testing. The intention of the CDC lipid standardization programs is to improve the accuracy and comparability of research-related testing, primarily through the Lipid Standardization Program (LSP), and to improve the accuracy of general clinical tests directly through the Cholesterol Reference Method Laboratory Network (CRMLN). There is strong evidence that laboratory performance on cholesterol testing has improved through standardization during the past 25 years, although it is difficult to say how much of the improvement has been due to the LSP and CRMLN.

**3.1.1 Accuracy of LSP Standardized Laboratories**

The goal of the LSP is to ensure that member labs exhibit consistent accuracy in lipid testing over time. Early lipid testing was subject to significant levels of error and bias, so initial efforts of the LSP focused on improving the accuracy of lipid testing through the development and establishment of reference testing methods. As standardization was achieved, the LSP focus turned to maintaining accuracy of lipid testing. Table 3-1 shows the percentage bias and coefficient of variation (CV) of LSP standardized labs (based on part III standardization maintenance surveys among newly enrolled labs that entered the program in each year) since electronic recordkeeping began in 1999. The results show that average bias levels have remained below 2% from 1999 through 2007 and have decreased by 0.7% percentage points over that time period. Labs are considered standardized if neither their percentage bias nor their CV exceeds 3%. Over that same period, less than 10% of surveys exhibited a bias of more than 3% except in 1999 and 2001, and no surveys yielded a CV of more than 3%. Note that the low number of observations may preclude drawing significant conclusions on the trend of "failing" labs over time.

**Table 3-1:** Accuracy of LSP-Standardized Labs, 1999–2007

**Year**	Observations	% Absolute Bias	% CV	% with Bias > 3% in Absolute Value	% with CV > 3%
1999	16	1.88	1.61	13%	0.00%
2000	4	1.42	1.54	0%	0.00%
2001	16	1.55	0.96	19%	0.00%
2002	36	1.24	1.07	6%	0.00%
2003	60	1.07	1.37	0%	0.00%
2004	8	1.24	1.34	0%	0.00%
2005	40	1.28	1.19	8%	0.00%
2006	24	1.24	1.21	4%	0.00%
2007	24	1.16	1.38	4%	0.00%

**3.1.2 Accuracy of Labs in CRMLN Clinical Laboratory Certification Program**

Although the LSP results demonstrate that standardization is being achieved among the limited number of research-oriented LSP standardized labs, the primary mechanism through which the CDC laboratory standardization programs may increase clinical testing accuracy is through the CRMLN. The CRMLN labs are intended to replicate CDC reference methods to extend the reach of standardization, most importantly through the manufacturer certification process. The manufacturer certification process allows manufacturers to calibrate their equipment and supplies against accuracy-based reference values. When used by clinical testing labs, the calibrated supplies will presumably increase the accuracy of clinical testing conducted by these labs. A resource for evaluating how well this program works is to look at data from clinical laboratories that participate in the CRMLN's Clinical Laboratory Certification Program. Table 3-2 shows the average percentage bias and average percentage CV among clinical laboratories participating in this program since 2000. Panel 3.2.a shows results for all labs applying to obtain or maintain certification, and Panel 3.2.b shows results for the subset of labs that obtained certification. As with the LSP standardized labs, the results show consistently low and improving bias and CV values.

**
Table 3-2. Accuracy of Clinical Laboratories Participating in CRMLN Certification, 2000–2009**

**3.2.a:** All Labs Applying to Obtain or Maintain Certification

**Year**	Observations	Average % Bias (Absolute Value)	Average % CV	% with Bias > 3% in Absolute Value	% with CV > 3%
2000	467	1.774	1.269	18%	3%
2001	431	1.641	1.197	15%	2%
2002	486	1.590	1.114	11%	1%
2003	435	1.411	1.083	11%	1%
2004	450	1.599	1.142	11%	1%
2005	443	1.509	1.224	12%	4%
2006	463	1.484	1.072	10%	2%
2007	441	1.512	1.129	10%	1%
2008	417	1.511	1.172	13%	1%
2009	408	1.498	1.151	12%	1%

**3.2.b:** Subset of Labs that Passed Certification

**Year**	0bservations	Average % Bias (Absolute Value)	Average % CV	% with Bias > 3% in Absolute Value	% with CV > 3%
2000	364	1.197	1.148	0.5%	1.1%
2001	356	1.196	1.117	0.6%	0.8%
2002	422	1.257	1.029	0.9%	0.0%
2003	371	1.049	1.005	0.3%	0.0%
2004	382	1.101	1.050	0.0%	0.3%
2005	367	1.147	1.091	0.3%	0.5%
2006	406	1.199	0.999	0.0%	0.2%
2007	390	1.232	1.090	0.8%	0.3%
2008	350	1.130	1.117	1.1%	0.3%
2009	351	1.080	1.095	0.3%	0.6%

**3.1.3 CAP Survey Results Show Improvement in Clinical Labs**

Although the above tables show that standardization is being achieved among labs participating in the LSP and CRMLN, these results do not directly reflect the accuracy of the many nonprogram labs that conduct patient clinical testing. Table 3-3 shows the results of CAP proficiency testing surveys for total cholesterol for major methods/instruments peer groups between 2000 and 2006. This period was selected because the CAP specimens were relatively free of matrix effects and CDC performed confirmatory testing on the materials used in the program. With the exception of 2001, we used the specimen in the year's A survey whose total cholesterol confirmatory value was closest to 200 mg/dL. Because the 2001 A survey was not available to us, we used the specimen in the B survey with a confirmatory value closest to 200 mg/dL.

**Table 3-3.** CDC Confirmed CAP Survey Results, 2000–2006

**Year**	Number of Labs	Number of Methods/ instruments	Mean	SD	Weighted Average Bias (% Absolute Value)	CV%	Number of Methods with bias >3% in Absolute Value	Number of Methods with CV > 3%
2000	4,731	34	208.1	5.2	1.18	2.5	4/34	6/34
2001	4,456	27	194.1	5.0	1.43	2.6	2/27	4/27
2002	4,330	26	188.8	4.6	0.87	2.5	2/26	6/26
2003	4,490	25	197.2	5.0	1.89	2.5	7/25	7/25
2004	4,156	23	196.8	4.8	1.35	2.4	3/23	4/23
2005	3,962	23	202.4	5.0	0.98	2.5	0/23	4/23
2006	4,080	21	201.8	4.8	0.69	2.4	1/21	2/21

Table 3-3 shows that bias and CV have remained consistently low and continue to show improvement at the method/instrument level, with the average absolute value of bias falling from 1.2% in 2000 to 0.7% in 2006, and the CV remaining unchanged. Four out of 34 methods/instruments had biases > 3% in absolute value in 2000 compared with 1 out of 21 in 2006; the corresponding figures for CV were 6 out of 34 in 2000 compared with 2 out of 21 in 2006.

Improvements in standardization have likely been much larger over a longer period, although the available data are not ideal for making long-term comparisons. Table 3-4 shows laboratory performance on CAP surveys in 1985 and 2009. These results show clear improvement in the bias (as measured by the percentage difference between a method/instrument mean and the all method/all instrument mean) and CV for each method/instrument. In 1985, about half of the method/instrument groups had biases greater than 3% in absolute value, and all but two method/instrument groups had CVs greater than 3%. In 2009, only one method/instrument group had a bias greater than 3% in absolute value, and only one group had a CV greater than 3%. However, the specimens used in 1985 and 2009 did not closely resemble patient samples and may have been subject to matrix effects (meaning that an instrument could perform well on patient samples but produce biased results on alterated, nonpatient samples). Nevertheless, the dramatic improvements shown in bias and in particular CV (which may be less susceptible to matrix effects) clearly suggest that standardization of labs occurred over this period.

**Table 3-4:** Laboratory Performance on CAP Proficiency Testing, 1985 and 2009

**Year**	Number of Labs	Number of Methods/ Instruments	Mean	SD	Weighted Average Bias (% Absolute Value)	Weighted average CV%	Number of Methods with Bias >3% in Absolute Value	Number of Methods with CV > 3%
1985	4,716	30	257.2	12.5	4.45	5.2	15/30	28/30
2009	4,770	20	203.0	4.4	1.67	2.2	1/20	1/20

Source: 1985—Laboratory Standardization Panel of the National Cholesterol Education Program, 1988; 2009—College of American Pathologists, 2009a. The underlying data are shown in Appendix Tables A-1 and A-2.

CAP launched its Accuracy Based Lipid (ABL) Survey in 2008 to eliminate or minimize matrix effects and provide better measures of the accuracy and harmonization of cholesterol testing. Results from the 2009 ABL (Table 3-5) suggest that almost all participating laboratories meet current NCEP standards for total cholesterol (total error within 9% of the target level). Most participating laboratories meet National Cholesterol Education Program (NCEP) standards for HDL cholesterol (total error within 13% of the target level), although the performance is not as strong as on total cholesterol. The ABL should be useful for identifying trends in laboratory accuracy as more years of data become available.

**Table 3-5.** Percentage of Laboratories Meeting NCEP Targets

	Total Cholesterol (within 10% of target)		

	**ABL-01**	**ABL-02**	**ABL-03**
Target	152.6 mg/dl	180.0 mg/dl	244.2 mg/dl
Labs	98.6%	100%	99.3%

	HDL Cholesterol (within 13% of target)		

	**ABL-01**	**ABL-02**	**ABL-03**

Target	33.9 mg/dl	56.8 mg/dl	49.3 mg/dl
Labs	77.4%	96.6%	91.8%

Source: College of American Pathologists, 2009b.

### 3.2 Medium-Term Outcomes

Increasing the accuracy of research and clinical testing will result in several medium-term outcomes, including improving clinical diagnosis rates and improving cholesterol-related research. Standardization of research testing has facilitated several important events, from the early research linking elevated total and LDL cholesterol to higher mortality, to more focused, clinical research on the efficacy of treatment and prevention interventions, including drugs and diet and exercise changes. Together, these findings have allowed for the creation of the ATP practice guidelines and provided the impetus for numerous public health campaigns targeted toward increasing physician and public awareness of the risks of high cholesterol and its modifiable risk factors.

**3.2.1 Improved Clinical Diagnosis Rates**

Better laboratory accuracy facilitates better diagnosis of persons with high cholesterol. If a laboratory produces biased cholesterol readings, some patients who truly need cholesterol reduction may not receive treatment, whereas other patients who do not need treatment may receive it. We used the data on bias from the method/instrument observations underlying Table 3-3 to estimate the percentage of patients whose total cholesterol would be misclassified in 2000 and 2006. We examined the ATP III cutoffs of 200 and 240 mg/dl to distinguish between desirable, borderline, and high total cholesterol. We used cholesterol information for U.S. adults from the 1988–1994 National Health and Nutrition Examination Survey (NHANES III), as reported in the ATP III report (ATP III, 2002), to calculate separate total cholesterol population distributions for men and women. For men, the data are roughly consistent with a normal distribution with mean 202 and variance 40.5. For women, the data are roughly consistent with a normal distribution with mean 206 and variance 43.5. Using the percentage bias for each individual laboratory method in 2000 and 2006 from the data underlying Table 3-3, we calculated the percentage of people misclassified relative to the "true" ATP-III distributions. For example, if the true distribution for males was distributed normally with a mean of 200 and variance of 10, then we would expect to find 50% of men classified as having desirable cholesterol (<200 mg/dl); however, if an individual laboratory method had 5% bias so that its distribution of cholesterol values was distributed normally with a mean of 210 and variance of 10, then too few men would be classified as having desirable cholesterol level and too many would be put into the borderline or high cholesterol categories.

Tables Table 3-6 and Table 3-7 show the percentage of adults whose total cholesterol would be correctly reported (so that a patient's reported value is the same as the true value, as highlighted in green) and the percentage that would be misclassified (so that the patient's reported value is not the same as the true value, as highlighted in red) in 2000 and 2006. The reduced bias in 2006 leads to fewer misclassifications. In 2000, a total of 3.9% of the male population would have been misclassified, including 0.5% of the population with true high cholesterol who would have been reported as having borderline cholesterol. In 2006, misclassifications reduced to 2.3% of the male population, including 0.2% of the population with true high cholesterol who would have been reported as having borderline cholesterol. Values for the female population exhibit the same trend toward fewer misclassifications.

If we conduct this same exercise using the 1985 and 2009 CAP proficiency testing survey data, the share of the population that would be misclassified is much larger (Tables 3-8 and 3-9) in 1985 with misclassifications falling dramatically by 2009. These results should be interpreted cautiously, however, because these specimens may have included matrix effects that distorted the true bias in patient samples.

**Table 3-6.** Percentage of Patients Misclassified in 2000 and 2006, Based on Total Cholesterol, Men

	True Desirable <200 mg/dl		True Borderline 200–239 mg/dL			True High >240 mg/dl	

	Reported as Desirable	Reported as Borderline	Reported as Desirable	Reported as Borderline	Reported as High	Reported as Borderline	Reported as High
True values	48.0%	—	—	34.6%	—	—	17.4%
2000	46.4%	1.6%	0.7%	32.7%	1.1%	0.5%	16.9%
2006	46.9%	1.1%	0.3%	33.5%	0.7%	0.2%	17.2%

**Table 3-7.** Percentage of Patients Misclassified in 2000 and 2006, Based on Total Cholesterol, Women

	True Desirable <200 mg/DL		True Borderline 200 – 239 mg/dL			True High >240 mg/dl	

	Reported as Desirable	Reported as Borderline	Reported as Desirable	Reported as Borderline	Reported as High	Reported as Borderline	Reported as High
True values	44.5%	—	—	33.8%	—	—	21.7%
2000	43.0%	1.5%	0.7%	31.9%	1.2%	0.5%	21.2%
2006	43.5%	1.0%	0.3%	32.7%	0.8%	0.2%	21.5%

**Table 3-8.** Percentage of Patients Misclassified in 1985 and 2009, Based on Total Cholesterol, Men

	True Desirable <200 mg/dl		True Borderline 200–239 mg/dL			True High >240 mg/dl	

	Reported as Desirable	Reported as Borderline	Reported as Desirable	Reported as Borderline	Reported as High	Reported as Borderline	Reported as High
True values	48.0%	—	—	34.6%	—	—	17.4%
1985	43.4%	4.6%	3.5%	27.5%	3.6%	2.1%	15.3%
2009	46.4%	1.6%	1.7%	31.8%	1.1%	1.0%	16.4%

**Table 3-9.** Percentage of Patients Misclassified in 1985 and 2009, Based on Total Cholesterol, Women

	True Desirable <200 mg/DL		True Borderline 200 – 239 mg/dL			True High >240 mg/dl	

	Reported as Desirable	Reported as Borderline	Reported as Desirable	Reported as Borderline	Reported as High	Reported as Borderline	Reported as High
True values	44.5%	—	—	33.8%	—	—	21.7%
1985	40.2%	4.3%	3.3%	26.8%	3.7%	2.3%	19.4%
2009	43.0%	1.5%	1.6%	31.0%	1.2%	1.1%	20.6%

**3.2.2 Practice Patterns**

High levels of cholesterol, including LDL cholesterol, were not definitively linked to increased risk of heart disease until the publication of the results of the Lipid Research Clinics Coronary Primary Prevention Trial in 1984 (Lipid Research Clinics Program, 1984). In 1985, NHLBI formed NCEP to organize public health efforts to reduce cholesterol-attributable heart disease. At the heart of these efforts are the clinical practice guidelines, the latest of which is the *Detection, Evaluation, and Treatment of High Blood Cholesterol in Adults* (ATP III, 2002).

To produce the practice guidelines, NCEP and its partners had to synthesize data from a number of sources to produce a comprehensive cholesterol control strategy. For example, epidemiological and clinical trials have demonstrated the harms of high cholesterol in the overall population and the degree of elevated risk among subpopulations. Other trials have assessed the efficacy of treatment to reduce high cholesterol. Finally, epidemiological surveys such as NHANES are necessary to identify actual cholesterol levels in the population. Combining the outcomes of such disparate studies is only possible when the cholesterol values of each study are directly comparable. To achieve comparability, the major cholesterol studies used to produce the practice guidelines have depended on LSP standardized labs to ensure accuracy and allow comparability.

**3.2.3 Cholesterol Awareness**

An important goal of public health programs focused on cholesterol is to increase awareness of the risks of high cholesterol among physicians and the general public. NCEP has focused efforts in two key areas: (1) improving clinical practice to increase the detection of high cholesterol and improve cholesterol treatment, and (2) increasing public awareness of the risks of high cholesterol to promote cholesterol-reducing lifestyle choices. By working with physician associations, continuing to refine the ATP guidelines, and conducting national conferences on cholesterol, NCEP continues to focus on physician education and training. NCEP has also focused on public health information campaigns, including the Know Your Number campaign to highlight the risks of high cholesterol among the general public and the Healthy People 2000 and Healthy People 2010 campaigns, which set defined targets for population cholesterol control.

NCEP efforts to improve physicians' understanding of the risks of cholesterol on CHD appear to have been largely successful. The NHLBI Cholesterol Awareness Surveys found that the number of patients who had ever had their cholesterol levels checked increased from 35% to 75% between 1993 and 1995 (NHLBI Cholesterol Awareness Surveys press release, December 1995). This survey also found that physicians had lowered the threshold for initiating cholesterol reduction treatment and were generally in compliance with the ATP guidelines. A CDC study using Behavioral Risk Factor Surveillance System data found that the proportion of people who reported having their blood cholesterol screened in the preceding 5 years increased from 67.6% in 1991 to 73.1% in 2003 (Saddlemire et al., 2005). However, recent evidence finds that lower rates of dietary and pharmacologic therapy initiation remain among certain physician groups, indicating that education efforts need to continue (Yarzebski, Bujor, & Goldberg, 2002).

As with the development of the clinical guidelines, public health information campaigns are ultimately the product of multiple and disparate sources of data on the risks of high cholesterol and the effectiveness of different treatment and prevention strategies; as with the formation of the practice guidelines, this is possible only when the data used are directly comparable due to the underlying accuracy of the cholesterol measurements. Thus, the CDC lipid standardization programs have played an important role in facilitating the research necessary to inform, guide, and bolster public health information efforts.

**3.2.4 Cholesterol-Lowering Drugs**

There have been clear improvements in drug therapies to reduce LDL cholesterol levels and/or increase HDL cholesterol levels in recent years (the LSP does not standardize LDL testing, although the CRMLN does; to estimate LDL levels, most U.S. laboratories use the Friedewald equation, which depends on total cholesterol, HDL cholesterol, and triglyceride measures that are standardized by both programs). In particular, the introduction and widespread adoption of statins has revolutionized cholesterol management. Currently, six statins (atorvastatin, fluvastatin, lovastatin, pravastatin, rosuvastatin, and simvastatin) are approved for use in the United States. These statins have been shown to reduce LDL cholesterol by 34% to 55%, with the most recently approved statins producing the largest reductions (*Senior Journal*, 2005). Other cholesterol-lowering drugs include ezetimide, nictonic acid, fenofibrate, and gemfibrozil.

The standardization of cholesterol measurement has played an important but difficult-to-quantify role in the development of cholesterol-lowering drugs. Cholesterol-lowering drugs are approved based primarily on their safety and their efficacy in lowering LDL cholesterol levels. To assess efficacy, it is necessary to accurately and reliably measure cholesterol levels. Standardization of cholesterol testing allows a large number of patients to be tested in large, multicenter clinical trials. Standardization also facilitates comparisons across trials and allows improvements in cholesterol to be assessed in the context of previous epidemiological studies showing the relationship between standardized cholesterol levels and clinical outcomes.

**3.2.5 Diet and Exercise**

In addition to pharmacological cholesterol reduction treatment, diet and exercise are important for ensuring reductions in cholesterol levels. On the basis of observational study findings, ATP III lists physical inactivity and an atherogenic diet (which generally includes high cholesterol) as major modifiable risk factors for high levels of LDL cholesterol and low levels of HDL cholesterol. The consumption of saturated fats and cholesterol has been falling since the early 1970s. In 1972, the average American consumed 355mg of cholesterol and 13.2g of saturated fat with a total energy intake of 1,983 kilocalories per day. By 1990, the cholesterol and fat intake measures had improved to 291mg of cholesterol and 12.6g of saturated fat with 2,199 kilocalories consumed per day (Ernst, Sempos, & Briefel, 1997). So while total caloric intake has markedly increased, cholesterol and saturated fat have decreased both in proportional and absolute levels. In the following decade, the proportion of calories from saturated fat continued to fall, although total cholesterol intake decreased only in men and actually increased by 11g per day in women (Carroll, Lacher, & Sorlie, 2005). However, the consumption of LDL cholesterol has decreased (Carroll, Lacher, & Sorlie, 2005). As with cholesterol medications, the evidence for the efficacy of lifestyle interventions to mitigate these risk factors came from clinical and epidemiological research, which, in most cases, benefited from increased accuracy due to the LSP.

### References

Adult Treatment Panel III (ATP III). (2002). *The third report of the National Cholesterol Education Program (NCEP) expert panel on detection, evaluation, and treatment of high blood cholesterol in adults (Adult Treatment Panel III). Final Report*. National Cholesterol Education Program, National Heart, Lung, and Blood Institute, National Institutes of Health. NIH Publication No. 02-5215.

Carroll, M. D., Lacher, D. A., & Sorlie, P. D. (2005). Trends in serum lipids and lipoproteins of adults, 1960–2002. *Journal of the American Medical Association, 294*(14), 1773–1781.

College of American Pathologists. (2009a). *Survey 2009: C-B chemistry/therapeutic drug monitoring, participant summary*. College of American Pathologists.

College of American Pathologists. (2009b). *ABL-A: Accuracy based lipid: Participant summary*. College of American Pathologists.

Ernst, N. D., Sempos, C. T., & Briefel, R. R. (1997). Consistency between U.S. dietary fat intake and serum total cholesterol concentrations: The national health and nutrition surveys. *American Journal of Clinical Nutrition, 66*(Suppl), 965S–9672S.

Laboratory Standardization Panel of the National Cholesterol Education Program. (1988). Current status of blood cholesterol measurement in the United States. *Clinical Chemistry*, *34*(1), 193–201.

Lipid Research Clinics Program. (1984). The lipid research clinics coronary primary prevention trial results, I. Reduction in incidence of coronary heart disease. *Journal of the American Medical Association,*
*251*(3), 351–364.

National Heart, Lung, and Blood Institute Cholesterol Awareness Surveys [press release]. Bethesda, Md: National Heart, Lung, and Blood Institute; December 4, 1995.

Saddlemire, A. E., Denny, C. H., Greenlund, K. J., Coolidge, J. N., Fan, A. Z., & Croft, J. B. (2005). Trends in cholesterol screening and awareness of high blood cholesterol: United States, 1991–2003. *MMWR, 54*(35), 865–870

*Senior Journal*. (2005). Latest statistics show 30 percent of seniors using statins in 2002. Retrieved October 15, 2009, from http://seniorjournal.com/NEWS/Health/5-10-11StatinUse2002.htm.

Yarzebski, J., Bujor, C. F., & Goldberg, R. J. (2002). A community-wide survey of physician practices and attitudes toward cholesterol management in patients with recent acute myocardial infarction. *Archives of Internal Medicine, 162*, 797–804.

## References

[1] (2002). Adult Treatment Panel III (ATP III). The third report of the National Cholesterol Education Program (NCEP) expert panel on detection, evaluation, and treatment of high blood cholesterol in adults (Adult Treatment Panel III). Final report.

[2] (2009). Death rates from heart disease by selected characteristics. The 2009 statistical abstract: the national data book, from Table 112: age-adjusted death rates by major causes: 1960 to 2005.

[3] Heron MP, Hoyert DL, Murphy SL, Xu JQ, Kochanek KD, Tejada-Vera B (2009). Deaths: final data for 2006. Natl Vital Stat Rep.

[4] Ford ES, Ajani UA, Croft JB, Critchley JA, Labarthe DR, Kottke TE (2007). Explaining the decrease in US deaths from coronary disease, 1980-2000. N Engl J Med.

[5] Capewell S, Hayes DK, Ford ES, Critchley JA, Croft JB, Greenlund KJ (2009). Life-years gained among US adults from modern treatments and changes in the prevalence of 6 coronary heart disease risk factors between 1980 and 2000. Am J Epidemiol.

[6] Myers GL, Cooper GR, Winn CL (1989). The Centers for Disease Control-National Heart, Lung and Blood Institute Lipid Standardization Program. An approach to accurate and precise lipid measurements. Clin Lab Med.

[7] Kimberly MM, Caudill SP, Cooper GR, Dasti M, Monsell E, Myers GL (2008). CDC's Lipid Standardization Program: assuring quality in epidemiologic studies for 50 years. J Clin Lipidol.

[8] Myers GL, Kimberly MM, Waymack PP, Smith SJ, Cooper GR, Sampson EJ (2000). A Reference Method Laboratory Network for Cholesterol: a model for standardization and improvement of clinical laboratory measurements. Clin Chem.

[9] Thienpont LM, Van Landuyt KG, Stöckl D, De Leenheer AP (1996). Four frequently used test systems for serum cholesterol evaluated by isotope dilution gas chromatography-mass spectrometry candidate reference method. Clin Chem.

[10] Wholey JS (1979). Evaluation — promise and performance.

[11] (1988). Current status of blood cholesterol measurement in clinical laboratories in the United States: a report from the Laboratory Standardization Panel of the National Cholesterol Education Program. Clin Chem.

[12] Stein EA, Myers GL (1995). National Cholesterol Education Program recommendations for triglyceride measurement: executive summary. Clin Chem.

[13] Warnick GR, Wood PD (1995). National Cholesterol Education Program recommendations for measurement of high-density lipoprotein cholesterol: executive summary. Clin Chem.

[14] Cleeman JI, Grundy SM, Becker D, Clark LT, Cooper RS, Denke MA (2001). Executive summary of the third report of the National Cholesterol Education Program (NCEP) expert panel on detection, evaluation, and treatment of high blood cholesterol in adults (Adult Treatment Panel III). JAMA.

[15] Grosse SD (2008). Assessing cost-effectiveness in healthcare: history of the $50,000 per QALY threshold. Expert Rev Pharmacoecon Outcomes Res.

[16] Braithwaite RS, Meltzer DO, King JT, Leslie D, Roberts MS (2008). What does the value of modern medicine say about the $50,000 per quality-adjusted life-year decision rule?. Med Care.

[17] Dockins C, Maguire K, Simon N, Sullivan M (2004). Value of statistical life analysis and environmental policy: a white paper.

[18] Murphy KM, Topel RH (2005). The value of health and longevity.

[19] Gallaher MP, Mobley LR, Klee GG, Schryver P (2004). The impact of calibration error in medical decision making.

